# Recommendations for the development of Egyptian human biobanking ethical guidelines

**DOI:** 10.12688/wellcomeopenres.16556.2

**Published:** 2021-03-19

**Authors:** Ahmed Samir Abdelhafiz, Calvin W. L. Ho, Teck Chuan Voo

**Affiliations:** 1 Department of Clinical pathology, National Cancer Institute, Cairo University, Cairo, 11796, Egypt; 2 Faculty of Law, The University of Hong Kong, Hong Kong, Hong Kong; 3 Centre for Biomedical Ethics, Yong Loo Lin School of Medicine, National University of Singapor, Singapore., Singapore

**Keywords:** Biobanking, Ethical guidelines, Egypt, Informed consent, data sharing

## Abstract

**Background:** The development of biobanks is associated with the emergence of new ethical challenges. In Egypt, several biobanks have been established, but there are no specific local ethical guidelines to guide their work. The aim of this study is to develop recommendations for the Egyptian human biobanking ethical guidelines, which take into consideration the specific cultural and legal framework in Egypt.

**Methods:** We searched the literature for available biobanking ethical guidelines. Six themes were the concern of search, namely; informed consent, data protection, return of results, sharing of samples and data, community engagement, and stakeholder engagement. If a document refers to another guideline, the new source is identified and the previous step is repeated.

**Results:** Ten documents were identified, which were analyzed for the themes mentioned above. Guidelines and best practices were identified, and then compared with the published documents about ethical, legal and social issues (ELSI) related to biomedical research in Egypt to reach best recommendations.

**Conclusions:** We have proposed, by way of recommendations, key characteristics that a national ethics framework in Egypt could have. On informed consent, the practice of broad consent may be harmonized among biobanks in Egypt. Clear policies on return of research results, training requirements and availability of genetic counseling could also be instituted through the national framework. Additionally, such a framework should facilitate community and stakeholders engagement, which is important to secure trust and build consensus on contentious issues arising from sample and data sharing across borders and commercialization, among other concerns.

## Introduction

In Egypt, eight human disease-based biobanks have been established over the past few years ^1^. These biobanks are distributed across six governorates (from the north to the south of Egypt), where they collect samples from different categories of patients, including cancer patients, liver patients, and heart disease patients ( Table 1). While established biobanks are at different stages of maturity, still others are being established. Some of these biobanks are affiliated to university hospitals or governmental research centers, and others are affiliated to non-governmental organizations (NGOs). The stage of development or maturity, affiliation of the biobank and its position in the hierarchy of the organization affect the policies and procedures of the biobank. For example, biobanks that are still building their inventory of samples and data repositories may not have clear policies on access and sharing. Biobanks affiliated with public universities and governmental organizations could also have issues with commercialization and collaboration with pharmaceutical companies, which may not be the case with biobanks affiliated with NGOs. As biobanking grows in Egypt, mutual trust must be strengthened and sustained among different stakeholders. While these biobanks have their own governance and ethics policies and operating procedures, the divergent standards, expectations and practices that have emerged present a number of challenges to appropriate stewardship of valuable biological resources, including more effective management and sharing of these materials and related data. Recently, a Clinical Research Law was enacted by the Egyptian parliament and approved by the Egyptian president to regulate clinical research conducted on humans ^2^. The law endorsed the establishment of a supreme council to review the ethics of clinical research. This council is entrusted with following up the implementation of the provisions of the law and taking the necessary actions if violation of any provisions occurs ^2^. The absence of specific guidelines has been identified as a major challenge by members of research ethics committees (RECs) in Egypt ^3^. Although the aforementioned law applies mainly to clinical research and clinical trials and is not specific to pre-clinical research, it forests out principles and standards for protection of participants and highlights the importance of consistency with generally accepted international ethical standards ^2^. Where applicable, relevant provisions of this law will be referred to. 

**Table 1.** A list of biobanks in Egypt.

Affiliation of the biobank	Organization hosting the biobank	Governorate	Patient categories
National Liver Institute, Menoufia University	University	Menoufia	Cancer and non-cancer
Children's Cancer Hospital 57357	NGO	Cairo	Cancer
National Cancer Institute, Cairo University	University	Cairo	Cancer
Faculty of Medicine Ain Shams Research Institute	University	Cairo	Cancer
South Egypt Cancer Institute	University	Assiut	Cancer
Shefaa Al Orman Oncology Hospital	NGO	Luxor	Cancer
MagdiYacoub heart foundation	NGO	Aswan	Non-cancer
Alexandria University	University	Alexandria	Cancer and non-cancer

* All biobanks are disease based, and all of them are non profit.

An increasingly diverse pool of biobanks and complex biobanking practices, along with a range of emerging ethical challenges (including issues related to unfair discrimination arising from sharing of different types of samples and data), may aggravate well-recognized ethical concerns (such as informed consent and privacy) ^4, 5^. First, it is not always clear which approach to consent-taking is ethically most appropriate for the type of biological material and data being collected for the purposes of primary and secondary research uses. Second, it is increasingly challenging to assure donors that their privacy and confidential information will be always secure. Third, there is growing complexity in the management of incidental and secondary findings, even while there is greater expectation on the part of potential donors that certain results should be returned to them ^6, 7^. Fourth, sample and data sharing across borders, ownership of tissue, and commercialization continue to raise concerns about equity and public trust ^5, 6^. In general, Egyptian biobanks face the same challenges as other biobanks, but some are especially onerous in low resource settings like Egypt, such as issues relating to sharing of samples or data and fair distribution of benefits. These concerns and challenges have contributed to the development of a number of international and professional ethical guidelines and best practices over the past 20 years ^6, 7^. Normative guidance include, among others, different versions of NCI Best Practices for Biospecimen Resources (NCI Best Practices), the International Society for Biological and Environmental Repositories (ISBER) best practices, the Organization for Economic Cooperation and Development (OECD) Guidelines for Human Biobanks and Human Genetic Databases ^6^. It is important to note that most RECs in Egypt use international research ethics guidelines, such as the Declaration of Helsinki, the Islamic Organization for Medical Sciences (IOMS), and guidelines of the Council for International Organizations of Medical Sciences (CIOMS) to review research protocols ^3^. While these documents provide general technical and ethical guidance that are widely accepted, differences in social, cultural, political and institutional conditions have resulted in divergent interpretations and practices. A major concern is the lack of uniform ethical standards and safeguards, which could impede appropriate sharing of materials and data and might have legitimized different turfs that have been formed. Moreover, many guidelines may be too general and do not provide adequate guidance in low resource research environments, with limited or no supportive legal, social and cultural infrastructure. 

At an institutional level, biobanks attempt to address these issues through the establishment of an effective governance system which ensures protection of participants, integrity, accountability, transparency, and trust while being dynamic and flexible at the same time ^8– 11^. A clear governance structure that is consistent with local and international guidelines and/or regulations has been recognized to be important ^8, 10, 12^. Like biobanks elsewhere, the biobanks in Egypt have established governance structures that provide guidance on technical and ethical issues, and policies for committees that look into a number of operational concerns. While these institutional initiatives are important, they cannot adequately address the growing number of ethical concerns or provide public assurance. Health-related research that biobanks enable and support is as much a social concern, as it is a scientific endeavour ^13^. Certain concerns that arise from the biobanking enterprise itself or from research that a biobank supports require societal level engagement, deliberation and resolution. A national ethics framework for biobanking could be a means of harmonizing divergent standards and practices among different biobanks operating within a jurisdiction, and drawing participation from a broader range of interested stakeholders into dialogue and deliberation. Recently, a national biobank network has been established to harmonize biobanking activities in Egypt. Several committees including ethics, quality, and accreditation are being established for this purpose. One important role of this network is to enhance communication, transparency and trust among the different stakeholders, including patients/participants and researchers in the institutions, hospitals, or organizations where biobanks are located, and with policymakers at the national level.

In this work, we argue for the establishment of a national ethics framework for biobanks in Egypt and suggest the themes that should be addressed therein. This framework could be of importance for the new biobank network, where it could be used for the development of local guidelines and best practices for biobanks and related practices at the national level.

The substance of this proposed framework is based on the existing body of international guidance and best practices. In the identification of these themes, we draw from the international and professional guidelines, while remaining sensitive to locally relevant issues and cultural differences. In other words, we have sought to identify ethical themes that apply to all biobanks in Egypt, with reference to international guidelines, and taking into consideration the specific cultural and legal framework in Egypt. It should be noted that although these themes apply mainly to Egypt, they are likely to be applicable to varying degrees to other developing countries in Africa and the Arab region due to similar infrastructure and/or cultural frameworks.

## Methods

The research questions that guided our policy review were as follows: (1) Which are the generally accepted transnational (or international) ethical guidance on biobanking and related data practices? (2) What are the main ethical norms or standards common to all transnational guidance documents, and applicable to all biobanks in Egypt? From our review of the literature published over the past ten years, we identified transnational ethical guidance on biobanking and related data practices (set out in Table 2), many of which were common points of reference by scholars. The literature search, which was carried out from January to March 2020, included Google, Google Scholar and PubMed. The search terms included the words "Biobanking ethics", "Biobanking ethical guidelines", "Biobanking guidelines". The terms were searched on these websites without adding any parentheses or quotation marks. Six main themes have been the concern of search and are also pertinent to the context of biobanking in Egypt, namely; informed consent, data protection, privacy and confidentiality, return of results and incidental findings, access to and sharing of samples and data and commercialization issues, community engagement and stakeholders engagement. These themes have been highlighted to be important in recent literature on biobanking and related data use for research purposes. They also underscore key processes of biobanks in Egypt, starting from the establishment of a biobank, through sample and data collection and distribution, as well as those that concern return of results and incidental findings. Any transnational or international document (as well as explanatory documents) on any of these themes and providing recommendations for them was included. However, documents that set out recommendations specific for a single country and cannot be applied in Egypt were excluded. General guidelines or documents on research ethics that are not directly applicable to biobanking were also excluded. 

**Table 2.** Relevant document/guideline/best practice related to biobanking ethical guidelines.

Document/Guideline/Best practice	Year issued	Type	Issued by	Themes covered	Relevance
OECD Guidelines on Human Biobanks and Genetic Research Databases ^8^.	2009	Guideline	Organization for Economic Cooperation and Development.	Informed consent Privacy Access and sharing Return of results	An international document that provides guidance for the establishment, governance, access, use of human biobanks.
Common Minimum Technical Standards and Protocols for Biobanks Dedicated to Cancer Research ^10^.	2017	Guideline	The International Agency for Research on Cancer (IARC)	Informed consent Privacy Access and sharing Return of results Community engagement Stakeholders engagement	An international document that includes guidelines and recommendations for biobanks. The based on evidence-based guidelines. The book includes sections on ELSI and sample sharing
WMA Declaration of Taipei on Ethical Considerations regarding Health Databases and Biobanks ^11^.	2016	Guideline	World Medical Association	Informed consent Privacy Access and sharing	An international document that covers the collection, storage and use of samples and data for research. In agreement with the Declaration of Helsinki, this document provides ethical principles for their use in Biobanks
Ethics and Governance Framework for Best Practice in Genomic Research and Biobanking in Africa ^12^.	2017	Guideline	H3Africa Working Group on Ethics.	Informed consent Access and sharing Return of results Community engagement	A regional document that provides a framework to provide an approach for best practice for biobanking in Africa
International Ethical Guidelines for Health-related Research Involving Humans ^13^.	2016	Guideline	Council for International Organizations of Medical Sciences (CIOMS) in collaboration with the World Health Organization (WHO)	Informed consent Privacy Access and sharing Community engagement Stakeholders engagement	An international document that provides internationally accepted ethical principles and how ethical principles should be applied, with attention to research in low- resource settings.
NCI Best Practices for Biospecimen Resources ^14^.	2016	Best Practices	Biorepositories and Biospecimen Research Branch National Cancer Institute	Informed consent Privacy Access and sharing Return of results Community engagement Stakeholders engagement	A national document that provides guidelines for biospecimen best practices, and supports adherence to ethical and legal requirements.
ISBER Best Practices: Recommendations for Repositories - 4th Edition ^15^.	2018	Best Practices	International Society for Biological and Environmental Repositories	Informed consent Privacy Access and sharing Return of results Community engagement	An international document that provides guidance in different aspects of biobanking base on the experience of different biobanking experts.
UK Biobank Ethics and Governance Framework ^16^.	2007	Guideline	UK Biobank	Informed consent Privacy Access and sharing	A local document from a biobank with many overseas collaborations; the document sets out the ethics and governance principles and mechanisms for the biobank and its collaborators.
Recommendation CM/Rec(2016)6 of the Committee of Ministers to member States on research on biological materials of human origin ‒ Explanatory Memorandum ^17^.	2016	Guideline	Council of Europe.	Informed consent Privacy Access and sharing Return of results	A regional document on research on biological materials of human origin that aims to protect the rights of individuals whose samples could be used in research project after collection and storage, while benefiting researchers from the access to biological materials.
Framework for Responsible Sharing of Genomic and Health- Related Data ^18^.	2014	Guideline	Global Alliance for Genomics and Health (GA4GH)	Informed consent Privacy Access and sharing	An international document that provides a framework to accelerate progress in human health through effective and responsible sharing of genomic and clinical data.

Potentially relevant papers and documents have been searched thoroughly for relevance to the subject. If a publication or a guideline refers to another guideline, the second source is identified and the previous step is repeated. To reach best recommendation, we also searched the literature for publications or regulations related to the ethical, legal, and social issues related to biomedical research or biobanking in Egypt. The search terms included "Research ethics in Egypt", "Clinical research law in Egypt", "Research ethics guidelines in Egypt", and "Biobanking in Egypt". Search results that did not apply to biobanking and related practices in Egypt were excluded. While specific and recent documents or regulations discussing these issues were included, general or old version of documents that have been updated were excluded. A possible limitation of our study is that the determination of ethical or normative themes that are relevant to the context of Egypt is based on the experience of one of the authors (ASA), as well as the authors’ understanding of the relevant laws and policies in Egypt. There may therefore be inherent biases or presumptions in our interpretation and/or understanding of the Egyptian context.

## Results

Ten relevant documents and six themes that relate to biobank and data governance were identified. Each document was analyzed for the guidelines stated for each of the six titles mentioned above. A list of the guidelines, themes covered in each of them, and their relevance to the current work is listed in Table 2. Guidelines and best practices were identified, and then compared with the published documents about ELSI related to biomedical research in Egypt to reach best recommendations ( Table 3). In the section that follows, we consider how a national framework that encompasses these themes can advance ethical practices (inclusive of responsible sharing of biological materials and related data) in Egypt.

**Table 3.** List of relevant documents/laws/publications related to medical ethics in Egypt.

Document/law/publication	Type
Clinical research law ^2^	Law
Sleem H, El-Kamary SS, Silverman HJ. Identifying structures, processes, resources and needs of research ethics committees in Egypt. BMC Med Ethics. 2010 Jun 28;11:12. doi: 10.1186/1472-6939-11-12. PMID: 20584332; PMCID: PMC2908628 ^3^.	Publication
Marzouk D., Abd El Aal W., Saleh A. Overview on health research ethics in Egypt and North Africa. Eur. J. Public Health. 2014;24(Suppl 1):87–91 ^19^.	Publication
Rashad, A. M., Phipps, F. M., & Haith-Cooper, M. (2004). Obtaining Informed Consent in an Egyptian Research Study. Nursing Ethics, 11(4), 394–399. https://doi.org/10.1191/0969733004ne711oa ^20^	Publication
Abdelhafiz, A.S., Sultan, E.A., Ziady, H.H. *et al.* What Egyptians think. Knowledge, attitude, and opinions of Egyptian patients towards biobanking issues. BMC Med Ethics 20, 57 (2019). https://doi.org/10.1186/s12910- 019-0394-6 ^24^	Publication
Abou-Zeid A, Silverman H, Shehata M, *et al.* Collection, storage and use of blood samples for future research: views of Egyptian patients expressed in a cross sectional survey. J Med Ethics. 2010;36(9):539–47. https://doi. org/1 0.1136/jme.2009.033100 ^25^.	Publication
El-Awa M.M. (2016) Confidentiality and Privacy in the Egyptian Legal System. In: Confidentiality in Arbitration. IusGentium: Comparative Perspectives on Law and Justice, vol 56. Springer, Cham ^26^.	Publication
Data protection law ^27^	Law

## Discussion

The quality of research in Egypt needs to be improved through funding, international collaboration, capacity building and sharing of scientific data. Medical research should be done in accordance to local and international laws and regulations pertaining to human subject research ^19^. In general, the laws in Egypt should not contradict the Quran and Islamic texts ^20^. According to the Egyptian constitution "“Islam is the state religion, and that principles of Islamic Jurisprudence are the main source of legislation" ^21^. Thus, it is important to consider religious issues while discussing the development of biobanking ethical guidelines in Egypt. Previous studies have shown that the establishment of research biobanks is allowed, and the issues of autonomy, confidentiality, beneficence and non-maleficence are respected in Islam ^20, 22, 23^. Interestingly, patients from the two main religions in Egypt, Islam and Christianity, who participated in a survey about biobanking did not think that there is a religious problem with donating samples for research in general ^24^. Consistent with these empirical findings, religion should not present a barrier to most types of biobanking activities and related research, although the guidelines should emphasize the need to be respectful of religious beliefs and practices. Based on the themes that were identified from the literature review, we examine how they may be applied in the context of Egypt.

### I. Informed consent

The aim of seeking consent is to inform potential participants about anticipated procedures, risks and benefits of participation and alternatives to participation so that they will be able to exercise voluntary choice on participation or contribution of biological material or related data to a biobank. As noted in the literature, this can be challenging if collected samples may be used for future and as yet unspecified research. This process may be even more challenging if future research use involves the generation and/or sharing of genomic data ^14, 28^.

As the literature shows, several types of consent may be used by a biobank; each has its advantages and disadvantages. These include, among others, specific consent (which ties consent to a specific research project), broad consent, dynamic consent (using technology to give participants the choice between broad consent or to approve one study at a time), and tiered consent (also called multilayer consent, where the participant may allow some uses of the samples only and renewal of consent is needed for other studies) ^6, 7, 10, 15^.

 A broad informed consent allows the use of samples in multiple future research projects. Unlike blanket consent in which there are no restrictions on use of samples, broad consent allows for potential future use in specified areas of research indicated to participants ^12, 13^. Broad consent should contain details about the biobank, type of collected data, protection of participants' rights, use, storage, access, possibility of re-contact, property rights, sharing conditions and benefit sharing, as wells as commercialization of the samples ^8, 11, 12, 14, 16^. Information on the right to withdraw, return of individual results, as well as risks especially if the uses include genome-wide association studies (GWAS) and gene sequencing studies, should also be provided ^14^. A broad consent mechanism covering these aspects is considered adequate to meet the informational needs of a prospective donor/participant, and is recognized to be acceptable for use by biobanks in the guidelines that we analyzed. This should be complemented by a robust ethics governance system, which usually includes a REC or institutional review board (IRB) where ethical requirements are concerned ^7, 10, 12, 13, 28^. RECs/IRBs oversee biobanking activities to ensure that the process meet these specifications ^13^. Moreover, REC/IRB must revise and approve all research proposals that may involve use of samples and data stored in the biobanks ^7, 13, 15^. If the scope of the submitted research proposals is beyond the scope of broad consent, new consent may be required by the REC/IRB, unless the requirement of consent is waived based on reasons such as minimal risk, necessity of using identifiable information, impracticability in obtaining consent, and public interest ^8, 10, 15^.

Research involving collection of biological samples from children should in general require heightened scrutiny due to their potential limited capacity to understand the concepts and implications of research ^29^. A balance between the benefits and risks is especially important in this case. In general, risk of physical harm associated with biobanks participation is lower than risk associated with clinical trial participation. In addition to the informed consent which should be provided by the legal representative, an assent may also be asked for according to the level of maturity and understanding of the child ^30^. Re-consent may be required when the child reaches adulthood ^30^. Another issue is return of research results, in which a biobank has to determine whether results will be returned or not, and whether the parents have the right to receive these kinds of results ^10, 17^. If the biobank is going to collect samples from children, clear policies and procedures about these issues should be in place before sample and data collection take place.

***Recommendation.*** The Egyptian Constitution states in Article 60 that: “It is not permissible to conduct any medical or scientific experiment on it (the human body) without the free and documented consent, in accordance with the established principles in the field of medical science, as regulated by law" ^21^. In addition, article 12 of the Clinical Research Law sets out specific requirements on the rights of research subjects or participants, including the right to getting a copy of the informed consent document, the right to withdraw at any time, and an obligation on the principal investigator to protect the confidentiality of personal data used in research. Chapter 10 of the legislation regulates the use of human samples for medical research and include provisions that forbid the use of such biological materials without obtaining the prior informed consent of the subject or his/her legal representative. The legislation also forbids storing left-over samples after the completion of medical research, or using them in future research or for any other purpose, without obtaining prior informed consent from the research participant and the approval of the Supreme Council ^2^.

Biobanks in Egypt generally recognize that informed consent is a critical prerequisite for human health related research. Currently, standard operating procedures (SOPs) in Egyptian biobanks give effect to these rights ^24^. While there is no data on public preference for a model of informed consent for biobanking in Egypt, broad informed consent is already used in most biobanks in Egypt. Other approaches, such as dynamic consent, may allow participants/donors to actively manage their preferences over time but this may not be feasible in some areas in Egypt due to limited access to the internet and related technology; at time of writing, less than 50% of Egyptians have access to the internet ^31^. The costs involved in establishing and maintaining a technological and data infrastructure to enable dynamic consent is likely to be a further obstacle. Apart from this, low literacy (especially among females and older people in Egypt) as shown in previous studies may limit the choice of consent models that may be effectively implemented in Egypt ^32^. A previous study showed that among Egyptian patients who agreed to participate in research, many of them preferred a consent model that limits the use of their samples to the disease being studied ^25^. A study by Labib *et al.* showed that most parents of children with cancer provide broad consent to biobanks for the research use of their children's biological materials and data ^33^. These findings provide some support for the adoption of broad consent and tiered consent models in some settings, where participants can choose between allowing the use of their samples for research concerning a particular type of disease (e.g. cancer) in general or only to specified types of research (e.g. lung cancer and other smoking related diseases). Although broad consent is generally an acceptable option, this model may not fully account for the preferences of individuals. Tiered consent may overcome these problems by providing potential participants with greater control over the future use of donated samples and data. Through responding to specific questions, these sample donors can specify acceptable levels of sample and data sharing ^34^. On the other hand, tiered consent may be time consuming and difficult to apply in some settings. For example, cancer patients usually suffer from psychological stress, especially during the first few visits to the hospital, where the biobank coordinators usually meet them and ask them to participate. Psychological stress could interfere with their ability to consider and discuss the different options. Significant illiteracy among certain individuals in Egypt may be another challenge, as they could have difficulty in understanding research terms and options.

While our national ethics framework could endorse the broad consent model, it will need to specify what basic information should be provided to prospective participants/donors and the means to promote understanding (such as the use of a simple Arabic glossary to explain the different terms that are commonly used in the informed consent), rights and interests of participants/donors that should be respected and promoted, and training and qualifications on ethics and communication that biobank operators and researchers must satisfy.

### II. Data protection, confidentiality, and privacy issues

Protection of privacy and confidentiality is one of the core elements for trust in biobanks ^11^. The whole process of sample collection, storage and distribution must respect the privacy and confidentiality of participants according to the local and international laws and regulations ^15^. Egyptian patients participating in a survey about biobanking highlighted the importance of privacy issues, and considered it as an important element of trust ^24^. Measures taken by the biobanks to protect privacy and confidentiality should be explained to potential donors during discussion of the informed consent ^16^.

Respecting privacy and maintaining confidentiality is generally recognized to be the duty of all staff members who have access to stored data. Biobank must have policies and appropriately designed information technology and data infrastructures for ensuring that personal data collected from participants are appropriately protected. Biobanks generally have SOPs to ensure that physical access, and access to personal data is restricted to persons in charge, and different levels of access are specified for operators ^24^. Regular audits on the data management system should be carried out on a regular basis to ensure the efficiency of the procedures taken by the biobank ^8^.

The literature has highlighted the need for training of staff members about issues in privacy and confidentiality, and technical issues related to these concerns ^17^. It is important to examine different means used to maintain privacy, to assess their effectiveness and to consider the possible need for re-identification for the purposes of re-contacting participants/contributors if they are agreeable to this ^7, 8, 13^. Researchers getting access to participants' samples and data will also be required to ensure that privacy and confidentiality safeguards will be maintained ^16^, and potential identification of participants should be highlighted and explained during submission of research protocols ^13^.

There is broad recognition that access to samples should be done through transparent, ethical and fair governance policies that allow maximum benefits to be derived from these limited resources ^14, 17^. In general, biobanks should not share samples or data with third parties for non-research purposes except when required and regulated by law ^8^. In the aforementioned survey about biobanking, about three quarters of Egyptian participants thought that law enforcement should have access to samples and data when necessary ^24^. Another survey conducted with a diverse group of participants in Saudi Arabia showed that most of them agreed that control of infectious diseases and access granted by a court order can be a reasonable justification for access to personal data without the consent of the participants/contributors concerned ^35^. There is at present a lack of clarity in Egypt over the probability and magnitude of potential harm or public interest that is necessary to justify a breach of privacy and/or confidentiality.

Protecting privacy and confidentiality may become more challenging with advancement in genome-sequencing technologies and advanced bioinformatics techniques. These include the risk of potential identification of the donor, as well as risks to their biological relatives if results show possibility of having a certain familial disease that may be stigmatizing ^8, 28, 36^. In addition, even if the biobank takes appropriate measures to protect privacy and confidentiality, there is possibility that data repositories may be broken into or stolen. Where research on rare diseases is concerned, the risk of re-identification may be greater.

***Recommendations.*** In general, the Egyptian constitution, legal system, as well as professional regulations highlight the importance of safeguarding privacy and confidentiality in medical as well as personal life ^2, 26^. Article 15 of the Clinical Research Law sets out the roles of the principal investigator and the study sponsor, if any, who are obliged not to publish any information, data or reports about the research except after its completion and getting a written approval for this purpose from the institutional committee, the Supreme Council, and after getting the written consent of the research participants if any specific information related to them is disclosed ^2^. A personal data protection law has been recently enacted by the Egyptian parliament ^37^, and the privacy principles are broadly similar to international guidelines on personal data protection. Although the law does not relate to clinical practice, some provisions relate to the protection of medical data. For example, Article 1 of identifies sensitive personal data as: "Data that disclose mental, physical or genetic health, biometric data, financial data, religious beliefs, political opinions, or security status. In all cases, children's data are considered sensitive personal data.", and Chapter 6 of the law is concerned with protection of such data and comprise provisions that highlight the importance of fulfilling the necessary data protection policies and procedures to avoid any breach or violation of personal data privacy ^27^.

Although personal data protection principles are mostly reflected in the SOPs of many biobanks in Egypt ^23^, there is currently no consistent approach among the biobanks in Egypt on addressing the risk of re-identification and how it can be explained during consent-taking.

While such a risk in genetics research is still low in Egypt as large-scale genomic studies are still limited, it is likely to become a concern in the foreseeable future and should be addressed in a national ethics framework. There may also be a need for regulatory oversight that involves regular audits of biobanks to ensure that measures to protect privacy and confidentiality are implemented and observed.

### III. Return of results and incidental findings

Biobanks usually use coding to link samples with associated data. This allows them to re-contact participants and to return individual or aggregate results, which should be carried out according to the wishes of the participants in the informed consent, where they should be informed about whether and how results will be returned ^10, 14, 17^.

Several types of results may be communicated with the individual and/or the community. General research results may be communicated with the public through websites or newsletters ^10, 15^. Individual test results may fall into two categories. First, there is initial general testing or retesting results (such as laboratory or radiological investigations). There are also research results which may be anticipated (within the goal of the study), or incidental findings (which may have potential health importance, but are not directly related to the original goal of research).

Return of results is associated with many challenges ^6, 12^. First, not every result should be returned to the participant. Returned results should be validated, clinically significant and could be associated with an action (e.g: The participant will have access to treatment) ^12, 18^. In other words, the findings should have high analytical validity, high clinical validity, and medical actionability ^38^. For example, if a genetic test is strongly predictive of an adverse clinical outcome, and has been validated through a second sample using a different or reference method, and possible intervention is available for participants in their particular contexts, these results should be returned ^38^. Second, individual counseling should be available for the participants and their families if the results include genetic findings, or if some family members don't want to know about results of familial diseases that may affect them ^3, 12, 30^.

Biobanks should have consistent and coherent policies and procedures for return of these results, where participants are informed that they can opt in or out of receiving genetic research findings ^38^, and should have a mechanism to integrate them with the healthcare system if this is approved in their SOPs ^8, 10, 15^. They should also be able to evaluate the validity of results, especially if they involve results of a complex (e.g. genetic) nature, and procedures to return results to participants based on agreed upon arrangements determined during consent-taking ^10^. Special considerations and arrangements apply when biological samples are collected from children and young persons, which includes processes to determine whether results should be returned or not, and the role of parents, guardians or communities concerned ^10, 17^.

***Recommendations.*** In the above mentioned study about the attitude of Egyptian patients towards biobanking, about 55% of participants thought that individual results derived from their tissue and of potential therapeutic value should be added to their medical record ^24^. In Egypt, return of genetic research results, including secondary and incidental findings is challenged by the difficulty in validating these results, and shortage of medical geneticists who can provide counseling for participants and their families. Although genetic counseling services are provided in Egypt, such services are still limited to a few centers all over the country. For example, El Hawary *et al.* described their experience in genetic counseling for families with children suffering from primary immunodeficiency disorders. Counseling included family education about the mode of inheritance of the disease, possibility of having an affected child, explanation of the available options for treatment, as well as the availability of prenatal diagnosis and preimplantation genetic diagnosis ^39^.

While return of results may not be a significant concern now, a national ethics framework should address these concerns and to ensure that both the research and healthcare communities are adequately equipped to meet these foreseeable challenges. Since Egypt is classified among low middle countries and biobanking practices are subject to similar resource constraints, the recommendations of Ethics and Governance Framework for Best Practice in Genomic Research and Biobanking in Africa, and H3Africa Guideline for the Return of Individual Genetic Research Findings may be the most suitable for Egypt ^12, 38^. A national framework should guide discussion between experts from the field of genetics, psychology and ethics to determine when and how return of genetic results, should take place. The need to train genetic counselors, and to expand genetic education of health professionals to communicate these findings to participants and their families should also be addressed in this framework ^40^. In the meantime, and until this happens, return of results should proceed with caution and should be limited to general laboratory or radiological investigations.

### IV. Access to and sharing of samples and data, benefit sharing and commercialization issues

Sharing of different types of samples and data is routine in international collaborative research. Biobanks play a central role in this process through being a custodian of these valuable resources. Results from research should be shared not only with the scientific community, but also with the biobanks as well. Such accumulation and dissemination of knowledge help to improve prevention, diagnosis and/or treatment of different diseases ^13, 16^. However, sharing of samples and associated data is associated with many challenges, including fair benefit sharing, intellectual property rights, as well as authorship over scientific publications. Ideally, these benefits should also be shared with the individual participants as well as the community concerned ^8, 18^.

Researchers' access to biological samples and data in a biobank should be done through fair and transparent processes based on coherent scientific and ethical criteria such as scientific merit ^14, 17^. Biotechnology and pharmaceutical companies, which play an important role in healthcare research through development of new biomedical products, require access to samples and data. Although commercialization of specimens and data is important for financial sustainability of biobanks ^41^, it raises questions about fair sharing of benefits, and is considered as a critical factor that may affect trust and participation ^42, 43^.

Biobanks should have consistent and fair policies about access to samples and data, and about commercialization and intellectual property rights ^8, 10, 11^. These policies should also indicate what provisions should be incorporated in material transfer agreements (MTA) and data transfer agreements (DTA), including the types and uses of samples and data that will be transferred, sharing of research results, citation or acknowledgment of the biobanks, patents and intellectual property rights ^8, 10^.

Although there is some guidance on data sharing in general, collaboration between high income and low-middle income countries (LMIC) raises a number of additional issues that require further deliberation and public engagement. LMIC play a growing role in research today. So, sample and data sharing should take into consideration the health needs of less developed countries, and the rights and interests of the communities participating in research should be protected ^11^.

Access to sample and data from these countries should be regulated by local committees (at the institution where the biobanks is based), or national committees, which must balance the potential benefits to science and humanity with fair benefits for the local community. This balance should be taken into consideration especially when commercialization issues and related benefits and burdens are discussed ^12^. The benefits for developing countries may include different forms of capacity building (e.g. training and developing research infrastructure), sharing in authorship of scientific publications, patents and sharing of intellectual property rights with local researchers, and providing access to commercial products at affordable prices for the local community ^12^. Achieving these goals is not easy, but a national ethics framework may help to support dialogue among the research community, international organizations, commercial entities, funders, sponsors and other interested stakeholders. In this respect, a recent Nuffield report on research in global health emergencies advocates authorship recognition for those who contribute data or samples for primary research, or whose data and samples are used for secondary research ^44^.

***Recommendations.*** The Clinical Research Law allows the import or export of any human samples for medical research only after approval of the Supreme Council has been obtained, and the requirements of national security must be considered ^2^. The law also prohibits any form of trading in human samples.

Egyptian patients have expressed concerns regarding sharing their samples with Western countries and pharmaceutical companies ^24, 25^. Egyptian physicians participating in a survey about biobanking were similarly concerned about commercialization, but data from this survey has not yet been published.

Due to sensitivity of the issues at the political and community levels, we recommend that sample and data sharing across Egyptian borders or with commercial entities should be governed by a legal framework and appropriate national-level ethical guidelines and processes. While this governance approach should protect public interests, it should not hinder research collaboration of scientific and social value. A national ethics framework may help to promote public discussion on how sample and data sharing and collaboration could support capacity building in Egypt. This framework could also help to inform provisions that should be included in MTA and DTA between biobanks with local and international researchers requiring access to samples and data, such as requirements relating to benefit sharing(e.g. who will be authors on publications, intellectual property rights, etc). Where commercialization is concerned, the national framework could provide guidance on when it should be allowed due to the novelty of biobanking and related data sharing in Egypt, and the need to build trust with stakeholders.

### V. Community engagement

The different processes of the biobank should be fully transparent, and showing respect is valuable for the successful conduct of research through ensuring acceptability and understanding the values of the proposed research ^13^. It is important to engage individuals and communities that have an interest in the research process ^12^. Engagement is a continuous process that should start from the development of the informed consent, through monitoring of the research process, till the dissemination of its results ^12, 13^. Research protocols and SOPs of biobanks should include plans for community engagement ^10, 12, 15^. Such plans should clearly identify the goals and processes for community engagement, and should allow the community to find out more about the biobank and, when possible, provide means to participate in the discussions and research use of stored materials and data ^13^. The plan should be implemented by the researchers and their sponsoring institutions, and should be reviewed at regular intervals ^12, 13, 18^.

Community engagement can take several forms; such as public forums, and inclusion of representatives from patient groups or the community ^10, 14^. This participation has mutual benefits to both parties: On one side, it will promote understanding on the part of community members and trust by providing the opportunity to talk about their needs and raise any concerns ^12, 13^. On the other side, this will inform biobank operators about the communities’ cultures and perceptions about different biobanks policies and practices, including sensitive issues ^12, 18^. Failure to secure trust may threaten the long-term viability of biobanks as individuals and communities refuse to contribute their materials or data ^13^.

***Recommendations.*** In Egypt, there are no clear models for public engagement in research. We believe that transparent and open public discussion about sensitive issues such as sample and data sharing and commercialization will help build public consensus on these issues. We recommend using social media as one of the platforms for community engagement as these platforms have shown effectiveness for communication about biobanking and health issues with different stakeholders ^1, 45^. Interestingly, growth of interest in health and medical research in Egypt accompanied the emergence of COVID-19 pandemic. We think that this could be a good time to start new initiatives of community engagement in research. 

### VI. Stakeholders engagement

Meaningful engagement of different stakeholders is important to ensure the long-term viability of biobanks ^10, 15^. Biobank stakeholders include patients, researchers, community leaders and representatives, regulatory authorities, government agencies, funders, as well as the general community. Transparency, effective communication and trust should also be promoted among these different stakeholders before the initiation of sample collection processes ^15^.

A biobank governance framework should support communication among the different stakeholders. This framework should include different institutional leaders to whom the biobanks manager reports ^15^, as well as oversight committees which oversee the process and support transparency and accountability ^10, 15^.

Researchers, as users of the biological materials and related data, should know about the existence of biobanks, and the resources that are maintained by them. Cost recovery models and service fees should be developed by biobanks in consultation with key stakeholders, reviewed regularly and adjusted as needed ^15^.

***Recommendations.*** In a study about their knowledge, perceptions, and attitude of about biobanking, Egyptian physicians reported limited knowledge about the existence of biobanks in Egypt. They also had concerns regarding broad consent and use of user fees by biobanks (Data not published). Taken together, we believe that these results represent limited engagement with these stakeholders. A national framework can help to promote engagement between biobanks and stakeholders (other than participants or contributors). Conferences, workshops, as well as social media could also be used for these purposes.

Development and implementation of biobanking ethics guidelines cannot proceed without the help and support of national and local RECs. It should be noted that although number and distribution of RECs is improving in Egypt ^20^, many of them suffer from administrative and financial limitations. Adoption of any guidelines should be done in parallel with improvement of RECs, through financial and administrative support and continuous training of their members ^3, 19^.

## Conclusions

We have identified some recommendations on themes that should be addressed in a national ethics framework on biobanking in Egypt. For informed consent, broad consent could be the most appropriate approach for biobanks in Egypt, although another possible option is tiered consent. In either cases, a national ethics framework can help to promote consistency among all the biobanks in Egypt and to set out the key governance requirements that are needed to support autonomous decision-making among donors/contributors, as well as public trust. This framework should also address current and anticipated challenges to safeguards on privacy and confidentiality, and to provide guidance on return of research results, which requires selection of the types of results to be returned, training for genetic of counselors and researchers, and better integration of the research and healthcare systems. Sample and data sharing across borders, and commercialization and intellectual property rights, draw a variety of issues that will require public engagement. More efforts are needed by the biobanking community to engage with different stakeholders, including the public. The proposed national ethics framework may help to enable public deliberation on the types of interests that should be protected by law, on benefit sharing and appropriate ethical and regulatory mechanisms that need to be established, including appropriate use of material and data transfer agreements.

## Data availability

### Underlying data

All data underlying the results are available as part of the article and no additional source data are required.
