# Supplementary material for: Recommendations for the development of Egyptian human biobanking ethical guidelines
Source: Wellcome Open Res. 2021 Mar 19;6:4. Originally published 2021 Jan 13. [Version 2] doi: 10.12688/wellcomeopenres.16556.2 (PMC8008429; doi:10.12688/wellcomeopenres.16556.2)
Supplement: Peer review history

# Peer review: Reviewer response for version 2

**DOI:** 10.21956/wellcomeopenres.18445.r43173

**Published:** 2021-03-26

**Authors:** Ciara Staunton

**Affiliations:** 1 Department of Law, Middlesex University, London, UK; 2 Institute for Biomedicine, Eurac Research, Bolzano, Italy

Thank you for your comments and the revised manuscript. Your responses have improved the paper. I just have 2 small further comments to make:

1. The Recommendation of the OECD Council on Health Data Governance (2017) is missing and may be useful to include.
2. On the structure, the sections labelled "recommendation" contain a lot of discussion. It would be better if these sections were solely focused on your recommendations. This would be better for readers looking to identify your recommendations. You may want to model it after the recommendations section on community engagement (although I would still encourage you to make your recommendations more clearer).

Is the work clearly and accurately presented and does it cite the current literature?

Partly

If applicable, is the statistical analysis and its interpretation appropriate?

Not applicable

Are all the source data underlying the results available to ensure full reproducibility?

Partly

Is the study design appropriate and is the work technically sound?

Yes

Are the conclusions drawn adequately supported by the results?

Yes

Are sufficient details of methods and analysis provided to allow replication by others?

Partly

Reviewer Expertise:

Legal and ethical issues associated with biobank research, genomic research and the use of health data.

I confirm that I have read this submission and believe that I have an appropriate level of expertise to confirm that it is of an acceptable scientific standard.

# Peer review: Reviewer response for version 1

**DOI:** 10.21956/wellcomeopenres.18243.r42427

**Published:** 2021-02-15

**Authors:** Ahmed El-Malky, Lana Shaiba

**Affiliations:** 1 Department of Pediatrics, Medical City King Saud University, Riyadh, Saudi Arabia; 2 King Saud University Medical City, King Saud University, Riyadh, Saudi Arabia

The work is clearly and accurately presented and does cite the current literature. Grammatical work, of developing argument and counter argument, and essay expression is accepted.

 The study design appropriate and the is work technically sound. I am very glad the authors wrote this essay. It is a well-written, needed, and useful summary of the current status of “data publication” from a certain perspective. The authors, however, need to be bolder and more analytical. This is an opinion piece, yet I see little opinion. A certain view is implied by the organization of the paper and the references chosen, but they could be more explicit.

 Sufficient details of methods and analysis provided to allow replication by others. The statistical analysis and its interpretation appropriate. The source data underlying the results available to ensure full reproducibility. The conclusions drawn adequately supported by the results

 I think this paper excellent and is an important addition to the literature. I really like the conceptualization of Recommendations for the development of Egyptian human biobanking ethical guidelines.

 There are minor corrections that do not affect the final decision: 

1. The methods chapter need some details about research question formulation. What is the problem are you trying to address by conducting this review? The research problem should be a structured and unambiguous question.
2. Define inclusion and exclusion criteria. Clearly state the criteria you will use to determine whether or not a study will be included in your search. Consider study populations, study design, intervention types, comparison groups, measured outcomes. 
3. Add a more detailed spreadsheet to describe all relevant data from each included study. It is recommended that you pilot your data extraction tool, to determine if other fields should be included or existing fields clarified.
4. Evaluate the risk of bias of included studies. Use a Risk of Bias tool (such as the Cochrane RoB Tool) to assess the potential biases of studies in regards to study design and other factors. Read the Cochrane training materials to learn about the topic of assessing risk of bias in included studies.

Is the work clearly and accurately presented and does it cite the current literature?

Yes

If applicable, is the statistical analysis and its interpretation appropriate?

Yes

Are all the source data underlying the results available to ensure full reproducibility?

Yes

Is the study design appropriate and is the work technically sound?

Yes

Are the conclusions drawn adequately supported by the results?

Yes

Are sufficient details of methods and analysis provided to allow replication by others?

Yes

Reviewer Expertise:

Public health and community medicine/ international health policies and healthcare CPGs.

We confirm that we have read this submission and believe that we have an appropriate level of expertise to confirm that it is of an acceptable scientific standard.

# Response

**Published:** 2021-03-10

**Authors:** Samir Abdelhafiz

**Affiliations:** National Cancer Institute, Egypt, Egypt

Dear Dr El-Malky, and Dr Shaiba,

 We are grateful for the instructive comments we received from you, which have helped to improve our manuscript. In addition, we also took the opportunity to update our manuscript on two recent developments. First, a clinical research law has been approved in Egypt. The law applies mainly to clinical research but is not specific to pre-clinical research. It provides guidance by setting out principles and standards for protection of participants and highlights the importance of consistency with generally accepted international ethical standards.  We have referred to applicable provisions of this law. Second, a national biobank network has been established to harmonize the activities of biobanks in Egypt. We have also highlighted this development in our manuscript. Kindly find our response to your comments below.

- The methods chapter needs some details about research question formulation. What is the problem are you trying to address by conducting this review? The research problem should be a structured and unambiguous question.  

Thank you for this helpful observation. We have clarified that the goal of our review is to identify key ethical norms or standards that are set out in generally accepted transnational guidance documents on biobanking and related data practices, and to further consider which of these norms or standards apply to the context of Egypt. We have clarified our research questions in the methodology section of the revised manuscript.

  

- Define inclusion and exclusion criteria. Clearly state the criteria you will use to determine whether or not a study will be included in your search. Consider study populations, study design, intervention types, comparison groups, measured outcomes.

Thank you for this helpful observation as well. We have clarified the inclusion and exclusion criteria, following from the research questions and goals the steered our project. As our research is not an intervention study, the conventional features of a research design (e.g. study population, intervention types, comparison groups, and outcome measures) do not apply to our study. Instead, a list of relevant policies is set out in Table 2.

- Add a more detailed spreadsheet to describe all relevant data from each included study. It is recommended that you pilot your data extraction tool, to determine if other fields should be included or existing fields clarified.

 A spreadsheet that includes data and quotations from different sources has been prepared.

- Evaluate the risk of bias of included studies. Use a Risk of Bias tool (such as the Cochrane RoB Tool) to assess the potential biases of studies in regards to study design and other factors. Read the Cochrane training materials to learn about the topic of assessing risk of bias in included studies.

Thank you for this recommendation, in response to which we have included in the manuscript a limitation of our review. We have explained that the determination of ethical or normative themes that are relevant to the context of Egypt is based on the experience of one of the authors (ASA), as well as our understanding of the relevant laws and policies in Egypt. As our study does not involve the assessment of fixed result from randomized clinical trials, the Cochrane Risk of Bias Tool is not applicable. However, we hope that in explicitly specifying the analytical vantage point that we have adopted as limitation, we acknowledge that there may be inherent bias in our interpretation and/or understanding of the Egyptian context, which should in turn be critically evaluated by the reader.

# Peer review: Reviewer response for version 1

**DOI:** 10.21956/wellcomeopenres.18243.r42134

**Published:** 2021-01-29

**Authors:** Ciara Staunton

**Affiliations:** 1 Department of Law, Middlesex University, London, UK; 2 Institute for Biomedicine, Eurac Research, Bolzano, Italy

I would like to thank the authors for the opportunity to review this paper. In the absence of a national framework for Egypt, the recommendations contained in this paper will be of the utmost importance.

 I do have a few points the authors may want to consider in revising this paper.

 I would suggest including much more background about the regulation of medical research more broadly in Egypt, research ethics review, any regulation of human tissues, and biobanks more specifically in the background. The new Data Protection Act is likely to have a great impact on biobanks and should be discussed here e.g. how will it impact research, are there special provisions for research, are the provisions unclear as they apply to research, etc. In South Africa, due to the uncertainty in the application of some of the principles of the Protection of Personal Information Act, the South African Academy of Sciences is developing a Code of Conduct for the use of health data in research. Is a similar Code needed in Egypt?

 The particular ELSI issues that arise in Egypt should be mentioned in the introduction also. Consider the literature in the region as it currently is very focused on these discussions in HICs. I think this discussion of the legal framework (or lack of) in Egypt and the ELSI issues that arise would be important in contextualising the paper. This is probably necessary as if you want to “to identify ethical themes that apply to all biobanks in Egypt, with reference to international guidelines, and taking into consideration the specific cultural and legal framework in Egypt” you do need to explain what these are.

 I would absolutely agree that there is a need for a national framework, but the justification could be stronger. You have alluded to these i.e. there are biobanks in Egypt that need governance, but make the case for public deliberation and engagement stronger. Perhaps link it to accountability and transparency. Many appear to be public bodies – are they biobanks in public institutions? If they are, perhaps you could reflect on that.

 Linked to this, the discussion on the policies of the governance of current biobanks needs to be a bit more robust. There are no links to the policies of these biobanks, their governance structures, and policies on related committees that you mention. How do you know what these policies contain and what are the differences in them? The differences in these frameworks could then provide a stronger justification for why you think there is a need for a national framework as there is currently no evidential basis for a lack of a harmonised process. Have you identified any gaps in them?

 There are some points in the methodology that would be good to clarify:

- In the methods section it seems to suggest that the themes guided the literature search, but in the results it seems that the themes came out of the search. Methods also initially seem to suggest that you were looking for policies only, but then seems to be that the process included getting other secondary sources.
- Related to that: “These themes have been highlighted to be important in recent literature on biobanking and related data use for research purposes.” – If the 6 themes to be looked at are coming from the literature, then the selection of these themes need to have a justification and link to the sources. Or is it the themes came from the guidelines?
- If you are comparing the guidelines with published ELSI related to biomedical research in Egypt then you probably should state what these papers are, how they were obtained and also provide a list of sources.
- When referring to the documents reviewed make it clear what are the guidelines and what is ELSI literature. Sometimes I wasn’t very sure.

In the results section it would be good to get clarity or further discussion on the following:

- What particular consent model will be affected by level levels of education?
- If previous research stated that Egyptians would like to limit their samples to the disease being studied, this would suggest that a very limited form of broad consent is acceptable and not in line with the definition of broad consent as you’ve presented. In reality, is it not tiered consent that would be best? What are the challenges with a tiered consent model in this context?
- Return of results: are medical geneticists considered to be able to provide counselling? Are there genetic counsellors in Egypt? The Ethics WG and other groups within H3Africa have also considered this issues in detail and this literature should be considered.
- Rather than saying what guideline may be more suitable, it would be of more benefit to have explicit recommendations for the national guideline.
- What impact does the Data Protection Act have on collection, access to and sharing (both within Egypt) and outside of Egypt’s borders?

Is the work clearly and accurately presented and does it cite the current literature?

Partly

If applicable, is the statistical analysis and its interpretation appropriate?

Not applicable

Are all the source data underlying the results available to ensure full reproducibility?

Partly

Is the study design appropriate and is the work technically sound?

Yes

Are the conclusions drawn adequately supported by the results?

Yes

Are sufficient details of methods and analysis provided to allow replication by others?

Partly

Reviewer Expertise:

Legal and ethical issues associated with biobank research, genomic research and the use of health data.

I confirm that I have read this submission and believe that I have an appropriate level of expertise to confirm that it is of an acceptable scientific standard, however I have significant reservations, as outlined above.

# Response

**Published:** 2021-03-10

**Authors:** Samir Abdelhafiz

**Affiliations:** National Cancer Institute, Egypt, Egypt

Dear Dr Staunton, 

 We are grateful for the instructive comments we received from you, which have helped to improve our manuscript. In addition, we also took the opportunity to update our manuscript on two recent developments. First, a clinical research law has been approved in Egypt. The law applies mainly to clinical research but is not specific to pre-clinical research. It provides guidance by setting out principles and standards for protection of participants and highlights the importance of consistency with generally accepted international ethical standards.  We have referred to applicable provisions of this law. Second, a national biobank network has been established to harmonize the activities of biobanks in Egypt. We have also highlighted this development in our manuscript. Kindly find our response to the reviewers’ comments below.

 Reviewer 1

- I would suggest including much more background about the regulation of medical research more broadly in Egypt, research ethics review, any regulation of human tissues, and biobanks more specifically in the background.

A recent clinical research law has been approved by the Egyptian president (after the manuscript was submitted). We added a brief introduction about the law in the introduction as follows: "Recently, a Clinical Research Law was enacted by the Egyptian parliament and approved by the Egyptian president to regulate clinical research conducted on humans. The law endorsed the establishment of a supreme council to review the ethics of clinical research. This council is entrusted with following up the implementation of the provisions of the law and taking the necessary actions if violation of any provisions occurs.  The absence of specific guidelines has been identified in the past as a major challenge by members of research ethics committees (RECs) in Egypt. Although the aforementioned law applies mainly to clinical research and clinical trials and is not specific to pre-clinical research, it forests out principles and standards for protection of participants  and highlights the importance of consistency with generally accepted international ethical standards.  Where applicable, relevant provisions of this law will be referred to.”

 Concerning regulation of biobanks and the ethics review in Egypt, we added the following about this point " It is important to note that most RECs in Egypt use international research ethics guidelines, such as the Declaration of Helsinki, the Islamic Organization for Medical Sciences (IOMS), and guidelines of the Council for International Organizations of Medical Sciences (CIOMS) to review research protocols” And the following reference was added :Sleem, H., El-Kamary, S.S. & Silverman, H.J. Identifying structures, processes, resources and needs of research ethics committees in Egypt. BMC Med Ethics 11, 12 (2010). https://doi.org/10.1186/1472-6939-11-12).

 We also added this paragraph about the recently established Egyptian biobank network. The corresponding author of this manuscript is a member in this network " Recently, a national biobank network has been established to harmonize biobanking activities in Egypt. Several committees including ethics, quality, and accreditation are being established for this purpose. One important role of this network is to enhance communication, transparency and trust among the different stakeholders, including patients/participants and researchers in the institutions, hospitals, or organizations where biobanks are located, and with policymakers at the national level.  .”

  

- The new Data Protection Act is likely to have a great impact on biobanks and should be discussed here e.g., how will it impact research, are there special provisions for research, are the provisions unclear as they apply to research, etc. In South Africa, due to the uncertainty in the application of some of the principles of the Protection of Personal Information Act, the South African Academy of Sciences is developing a Code of Conduct for the use of health data in research. Is a similar Code needed in Egypt?

The law is not directly related to medical research. However, we mentioned it in the context of our work since it is related to the general issue of data protection. The Clinical Research Law is more concerned with protection of privacy and confidentiality in the context of medical research. We mentioned that in the following paragraph “In addition, article 12 of the Clinical Research Law sets out specific requirements on the rights of research subjects or participants, including the right to getting a copy of the informed consent document, the right to withdraw at any time, and an obligation on the part of the principal investigator to protect the confidentiality of personal data used in research.” We also added some details about data protection from the recently approved clinical research law " Article 15 of the Clinical Research Law sets out the roles of the principal investigator and the study sponsor, if any, who are obliged not to publish any information, data or reports about the research except after its completion and getting a written approval for this purpose from the institutional committee, the Supreme Council, and after getting the written consent of the research participants if any specific information related to them is disclosed".

 We also added more details about the data protection law as follows "Although the law does not relate to clinical practice, some provisions relate to the protection of medical data. For example, Article 1 of identifies sensitive personal data as: "Data that disclose mental, physical or genetic health, biometric data, financial data, religious beliefs, political opinions, or security status. In all cases, children's data are considered sensitive personal data.", and Chapter 6 of the law is concerned with protection of such data and comprise provisions that highlight the importance of fulfilling the necessary data protection policies and procedures to avoid any breach or violation of personal data privacy."

- The ELSI issues that arise in Egypt should be mentioned in the introduction also. Consider the literature in the region as it currently is very focused on these discussions in HICs. I think this discussion of the legal framework (or lack of) in Egypt and the ELSI issues that arise would be important in contextualising the paper. This is probably necessary as if you want to “to identify ethical themes that apply to all biobanks in Egypt, with reference to international guidelines, and taking into consideration the specific cultural and legal framework in Egypt” you do need to explain what these are.

We think that the issue of trust is the major issues facing the growing biobanking field in Egypt. We added the following statement in the introduction " As biobanking grows in Egypt, mutual trust must be strengthened and sustained among different stakeholders”

 We already mentioned the issue about religion and how it could impact biobanks. This is an important issue in Arab/Islamic countries. 

 Concerning the legal framework, we included some articles of the newly approved clinical research law, and how it applies in some points to biobanking.

 Otherwise, challenges faced by biobanks are quite similar to other parts in the world. We added the following statement to clarify this point " In general, Egyptian biobanks face the same challenges as other biobanks, but some are especially onerous in low resource settings like Egypt, such as issues relating to sharing of samples or data and fair distribution of benefits.”

- I would absolutely agree that there is a need for a national framework, but the justification could be stronger. You have alluded to these i.e., there are biobanks in Egypt that need governance, but make the case for public deliberation and engagement stronger. Perhaps link it to accountability and transparency. Many appear to be public bodies – are they biobanks in public institutions? If they are, perhaps you could reflect on that.

Based on the previous point, where we stated that a major issue with biobanking in Egypt is trust, we think that these recommendations can help resolve this issue through the recently developed biobank network as we explained earlier in the following paragraph " Recently, a national biobank network has been established to harmonize biobanking activities in Egypt. Several committees including ethics, quality, and accreditation are being established for this purpose. One important role of this network is to enhance communication, transparency and trust among the different stakeholders, including patients/participants and researchers in the institutions, hospitals, or organizations where biobanks are located, and with policymakers at the national level. 

 In this work, we argue for the establishment of a national ethics framework for biobanks in Egypt and suggest the themes that should be addressed therein. This framework could be of importance for the new biobank network, where it could be used for the development of local guidelines and best practices for biobanks and related practices at the national level. "

  

- Linked to this, the discussion on the policies of the governance of current biobanks needs to be a bit more robust. There are no links to the policies of these biobanks, their governance structures, and policies on related committees that you mention. How do you know what these policies contain and what are the differences in them? The differences in these frameworks could then provide a stronger justification for why you think there is a need for a national framework as there is currently no evidential basis for a lack of a harmonized process. Have you identified any gaps in them?

There are no published data about the governance of these biobanks. However, the corresponding author of this manuscript participated in interviews with different biobank managers in Egypt as a part of another research project that aimed at evaluating stakeholders’ views in the Arab region, including Egypt, about biobanking (Data not published). These interviews showed that there is lack of harmonization about policies related to some issues such as collaboration with pharmaceutical companies and commercialization. We mentioned in the current manuscript that biobanks in Egypt are are in different stages of maturity, so some of them still don't have clear policies for access and sharing. We clarified this in the introduction as follows " While established biobanks are at different stages of maturity, still others are being established. Some of these biobanks are affiliated to university hospitals or governmental research centers, and others are affiliated to non-governmental organizations (NGOs). The stage of development or maturity, affiliation of the biobank and its position in the hierarchy of the organization affect the policies and procedures of the biobank. For example, biobanks that are still building their inventory of samples and data repositories may not have clear policies on access and sharing. Biobanks affiliated with public universities and governmental organizations could also have issues with commercialization and collaboration with pharmaceutical companies, which may not be the case with biobanks affiliated with NGOs. As biobanking grows in Egypt, mutual trust must be strengthened and sustained among different stakeholders "

 Another point that indicates the need of harmonization is the development of the Egyptian biobank network. The network which includes all biobank managers as well as other stakeholders aims mainly at harmonization of biobanking activities in Egypt.

  

- There are some points in the methodology that would be good to clarify: In the methods section it seems to suggest that the themes guided the literature search, but in the results it seems that the themes came out of the search. Methods also initially seem to suggest that you were looking for policies only, but then seems to be that the process included getting other secondary sources.  

Thank you for these helpful observations on the methodology. Our literature review focused on transnational policies that apply to biobanking and related data practices, but we did also include some secondary sources that helped to clarify or explain the applicability of these policy provisions to biobanking in Egypt. From the review, common themes were identified, and out of these themes, we focused on those that are relevant in guiding policy development in Egypt. We have clarified our methodology in the manuscript.

  

- Related to that: “These themes have been highlighted to be important in recent literature on biobanking and related data use for research purposes.” – If the 6 themes to be looked at are coming from the literature, then the selection of these themes need to have a justification and link to the sources. Or is it the themes came from the guidelines?

Thank you for observation, which is helpful. We have clarified that our review focused on transnational policies that are set out in Table 2, and on themes that are applicable to biobanking and related data practices in the Egyptian context.

  

- If you are comparing the guidelines with published ELSI related to biomedical research in Egypt then you probably should state what these papers are, how they were obtained and also provide a list of sources.

 

 We described how these sources were obtained in the methods as follows " we also searched the literature for publications or regulations related to the ethical, legal, and social issues related to biomedical research or biobanking in Egypt. The search terms included "Research ethics in Egypt", "Clinical research law in Egypt”, "Research ethics guidelines in Egypt", and "Biobanking in Egypt". Search results that did not apply to biobanking and related practices in Egypt were excluded. While specific and recent documents or regulations discussing these issues were included, general or older versions of documents that have been updated were excluded.”

 A list of these documents was added in table 3.

  

- When referring to the documents reviewed make it clear what are the guidelines and what is ELSI literature. Sometimes I wasn’t very sure in the results section it would be good to get clarity or further discussion on the following.

We added a column about the document type to table 2.

  

- What particular consent model will be affected by level levels of education?

There is no published data about this, and we stated that in the discussion "While there is no data on public preference for a model of informed consent for biobanking in Egypt, broad informed consent is already used in most biobanks in Egypt. Other approaches, such as dynamic consent, may allow participants/donors to actively manage their preferences over time but this may not be feasible in some areas in Egypt due to limited access to the internet and related technology; at time of writing, less than 50% of Egyptians have access to the internet" And later on " Apart from this, low literacy (especially among females and older people in Egypt) as shown in previous studies may limit the choice of consent models that may be effectively implemented in Egypt."

  

- If previous research stated that Egyptians would like to limit their samples to the disease being studied, this would suggest that a very limited form of broad consent is acceptable and not in line with the definition of broad consent as you’ve presented. In reality, is it not tiered consent that would be best? What are the challenges with a tiered consent model in this context?

Although the study by Abou-Zeid et al. stated that consent model that limits the use of their samples to the disease being studied, other studies show the acceptability of participants to broad consent. We added a reference for this as follows" A study by Labib et al. showed that most  parents of children with cancer provide broad consent to biobanks for the research use of their children's biological materials and data". Another clue for the acceptability of broad consent is the fact that it is already adopted by most biobanks in Egypt, with no reported problems.

 However, we agree that each type of consent has its own advantages. We added a reference about the value of tiered consent and discussed challenges associated with its use as follows " Although broad consent is generally an acceptable option, this model may not fully account for the preferences of individuals. Tiered consent may overcome these problems by providing potential participants with greater control over the future use of donated samples and data. Through responding to specific questions, these sample donors can specify acceptable levels of sample and data sharing.33 On the other hand, tiered consent may be time consuming and difficult to apply in some settings. For example, cancer patients usually suffer from psychological stress, especially during the first few visits to the hospital, where the biobank coordinators usually meet them and ask them to participate. Psychological stress could interfere with their ability to consider and discuss the different options. Significant illiteracy among certain individuals in Egypt may be another challenge, as they could have difficulty in understanding research terms and options.”   

- Return of results: are medical geneticists considered to be able to provide counselling? Are there genetic counsellors in Egypt? The Ethics WG and other groups within H3Africa have also considered this issue in detail and this literature should be considered.
  ​​​​​​​

Yes, there are genetic counselors in Egypt, but their number is limited, and they work in a few centers. We added a reference about the experience of genetic counseling in Cairo University as follows “Although genetic counseling services are provided in Egypt, such services are still limited to a few centers all over the country. For example, El Hawary et al. described their experience in genetic counseling for families with children suffering from primary immunodeficiency disorders. Counseling included family education about the mode of inheritance of the disease, possibility of having an affected child, explanation of the available options for treatment, as well as the availability of prenatal diagnosis and preimplantation genetic diagnosis.”

 We have also included H3Africa Guideline for the Return of Individual Genetic Research Findings as a reference as follows” In other words, the findings should have high analytical validity, high clinical validity, and medical actionability. For example, if a genetic test is strongly predictive of an adverse clinical outcome, and has been validated through a second sample using a different or reference method, and possible intervention is available for  participants in their particular contexts, these results should be returned”

- Rather than saying what guideline may be more suitable, it would be of more benefit to have explicit recommendations for the national guideline. ​​​​​​​

Thank you for this recommendation. We have indeed argued that it will be helpful to have a consistent set of ethical guidance for all biobanks, although such a guideline need not be ‘national’, in that it could be collaboratively developed and adopted by the Biobanks’ Network. Such a bottom-up approach could complement the top-down regime established under the newly enacted Clinical Research Law.

- What impact does the Data Protection Act have on collection, access to and sharing (both within Egypt) and outside of Egypt’s borders? 

 Since the law is not directly related to medical data, we do not know if the law will affect the process of collection, access or sharing. However, we included the chapters/articles that could be related to medical data as follows “Although the law does not relate to clinical practice, some provisions relate to the protection of medical data. For example, Article 1 of identifies sensitive personal data as: "Data that disclose mental, physical or genetic health, biometric data, financial data, religious beliefs, political opinions, or security status. In all cases, children's data are considered sensitive personal data.", and Chapter 6 of the law is concerned with protection of such data and comprise provisions that highlight the importance of fulfilling the necessary data protection policies and procedures to avoid any breach or violation of personal data privacy."
